# AAV Gene Augmentation of Truncated Complement Factor H Differentially Rescues Ocular Complement Dysregulation in a Mouse Model

**DOI:** 10.1167/iovs.64.10.25

**Published:** 2023-07-20

**Authors:** Daniel Grigsby, Mikael Klingeborn, Una Kelly, Lindsey A. Chew, Aravind Asokan, Garth Devlin, Sharon Smith, Lisa Keyes, Adrian Timmers, Abraham Scaria, Catherine Bowes Rickman

**Affiliations:** 1 Department of Ophthalmology, Duke University School of Medicine, Durham, North Carolina, United States; 2 McLaughlin Research Institute, Great Falls, Montana, United States; 3 Department of Cell Biology, Duke University School of Medicine, Durham, North Carolina, United States; 4 Departments of Surgery, Molecular Genetics and Microbiology, and Biomedical Engineering, Duke University School of Medicine, Durham, North Carolina, United States; 5 Applied Genetic Technologies Corporation, Alachua, Florida, United States; 6 Pfizer, Morrisville, North Carolina, United States; 7 Editas Medicine, Cambridge, Massachusetts, United States

**Keywords:** complement, complement factor H (CFH), age-related macular degeneration (AMD), retinal pigmented epithelium, gene therapy

## Abstract

**Purpose:**

Complement dysregulation in the eye has been implicated in the pathogenesis of age-related macular degeneration (AMD), and genetic variants of complement factor H (CFH) are strongly associated with AMD risk. We therefore aimed to untangle the role of CFH and its splice variant, factor H-like 1 (FHL-1), in ocular complement regulation derived from local versus circulating sources. We assessed the therapeutic efficacy of adeno-associated viruses (AAVs) expressing human FHL-1 and a truncated version of CFH (tCFH), which retains the functional N- and C-terminal ends of the CFH protein, in restoring the alternative complement pathway in *Cfh*^–/–^ mouse eyes and plasma.

**Methods:**

Using *Cfh*^–/–^ mice as a model of complement dysregulation, AAV vectors expressing tCFH or FHL-1 were injected subretinally or via tail vein, and the efficacy of the constructs was evaluated.

**Results:**

Following subretinal injections, tCFH expression rescued factor B (FB) retention in the eye, but FHL-1 expression did not. By contrast, both constructs restored FB detection in plasma following tail vein injections. Both tCFH and FHL-1 proteins accumulated in the posterior eyecup from the circulation following liver transduction; however, neither was able to significantly regulate local ocular complement.

**Conclusions:**

Our findings demonstrate that the C-terminus of human CFH is necessary for complement regulation in the murine eye. Furthermore, exogenous CFH must be synthesized locally to maximize complement regulation in the retina. These findings establish a critical foundation for development of CFH augmentation-based gene therapies for the eye.

Age-related macular degeneration (AMD) is a leading cause of vision loss in elderly populations in industrialized nations.^1^ The incidence of AMD increases dramatically with age,^2^^,^^3^ and this disease burden is expected to increase as the world's population ages.^1^ The first signs of AMD include thickening of Bruch's membrane (BrM), alterations in the retinal pigment epithelium (RPE) such as pigmentation changes, and accumulation of lipid and protein deposits known as drusen in BrM.^4^ Late-stage disease is characterized by either abnormal blood vessel growth and scarring in the neovascular or wet form or by atrophy of the RPE and photoreceptors in the non-neovascular dry form, geographic atrophy.^5^ Although the neovascular form of AMD has treatment options in the form of anti-vascular endothelial growth factor therapies,^6^^,^^7^ there are currently no therapies for intermediate AMD, and only one approved treatment exists for geographic atrophy.^8^ Thus, a better grasp of the pathophysiological and molecular mechanisms of the disease is needed to design treatments.

AMD is a complex disease, in which aging, environmental, and genetic factors contribute to its development and progression.^3^ Strong evidence based on biochemical, genetic, and cell biological studies implicates the alternative pathway of complement (APC) in AMD pathogenesis.^9^^–^^17^ In particular, the complement factor H (*CFH*) gene, where a nucleotide change results in a tyrosine (Y) to histidine (H) change in the short consensus repeat (SCR) 7 at amino acid (AA) 402 (Y402H) (Fig. 1A), dramatically increases the risk of AMD.^18^^,^^19^ The APC is an arm of the complement system and consists of a series of proteolytic cleavages that amplify the initial signal, and runaway activation can occur without negative regulators, of which CFH is the major soluble inhibitor.^20^^,^^21^ The complement system can be activated by several pathways that all converge on the protein complement component 3 (C3). The APC is characterized by formation of the C3bBb C3-convertase, and this pathway can be initiated by the spontaneous cleavage of C3 or through cleavage of C3 by C3-convertases from the classical or lectin pathways; the alternative pathway serves to amplify the signal of these other pathways.^22^ In AMD, the APC is further implicated genetically by the identification of AMD risk variants in other complement genes such as factor B (*CFB*), complement component 3 (*C3*), and factor I (*CFI*)^9^^,^^15^^–^^17^; like *CFH*, both *CFB* and *CFI* are unique to the APC. Variants in these genes are associated with disease risk and result in increased complement activation.^9^^,^^23^ *CFH* variants, especially the Y402H polymorphism, are common in the population and contribute to a large fraction of the population attributable risk for developing AMD.^19^^,^^24^

**Figure 1.**
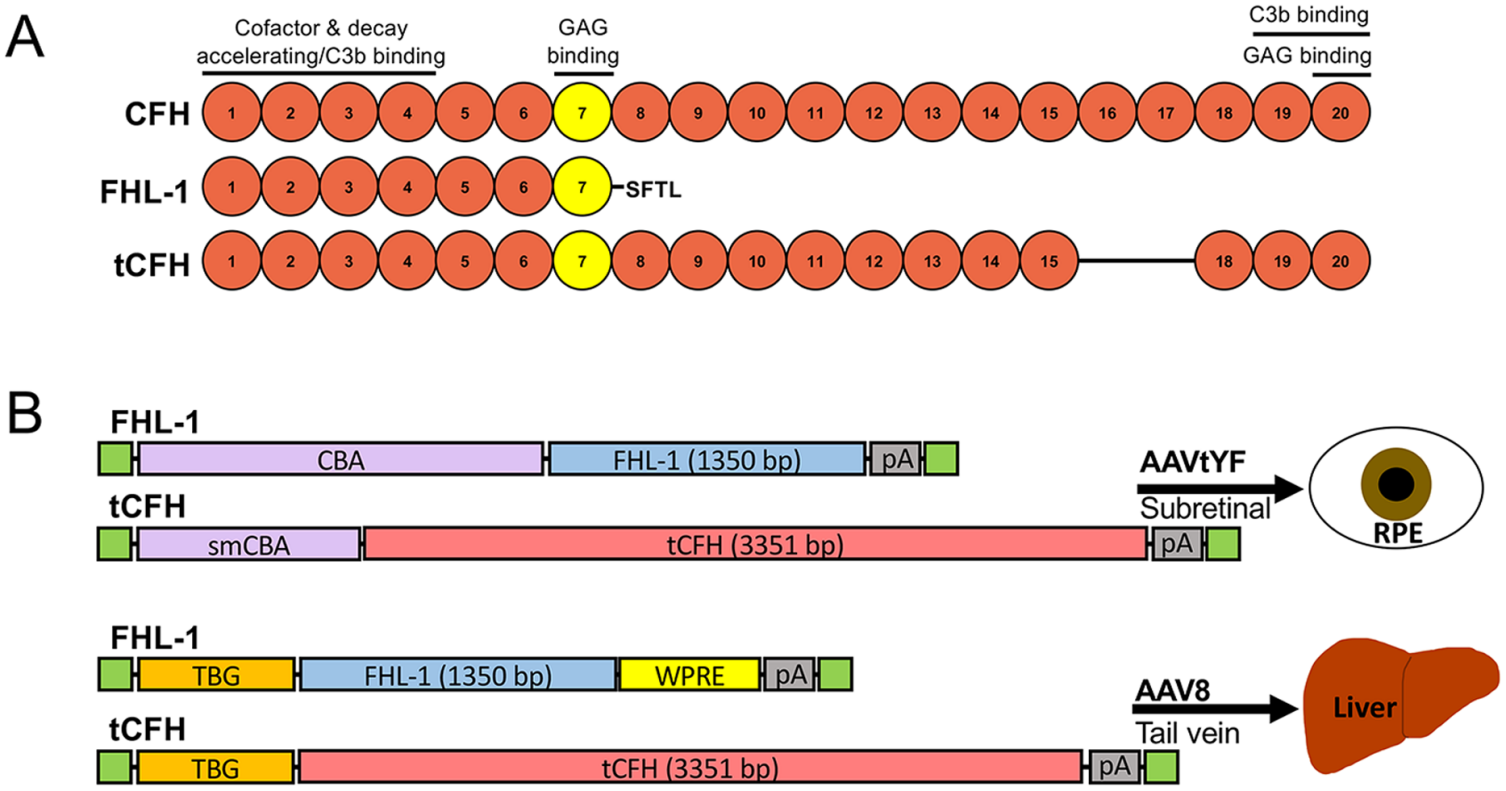
Complement factor H (CFH) constructs. (**A**) Diagram of short consensus repeats (SCRs) of human CFH, FHL-1, and tCFH proteins and function and ligand binding regions of SCRs. Full-length CFH is 1231 amino acids and 139 kDa. FHL-1, the human splice variant of CFH, consists of the first seven SCRs plus four unique amino acids Ser-Phe-Thr-Leu (SFTL) and is a total of 449 amino acids and 51 kDa. The truncated tCFH lacks SCRs 16 and 17 and is 1116 amino acids and 126 kDa. The Y402H variant is in SCR 7 (*yellow*). (**B**) Diagram of expression cassettes inserted into the AAV capsids for either subretinal or tail vein injection. A chicken β actin (CBA) promoter was used to drive nonspecific ocular expression, and constructs were packaged in an AAVtYF capsid for subretinal injection. smCBA is a smaller version of the CBA promoter, necessitated by the insert size constraints of AAV and the length of the tCFH construct. The thyroxine binding globulin (TBG) promoter was used to drive liver-specific expression, and constructs were packaged in an AAV8 capsid for tail vein injection. The *green boxes* represent the flanking inverted terminal repeats. WPRE, woodchuck hepatitis virus post-transcriptional regulatory element; pA, poly-adenylation signal.

The genetic association between *CFH* and AMD represents one of the most replicated genetic variants associated with AMD risk.^10^^–^^13^ Numerous subsequent studies have focused on elucidating the functional differences between the normal Y402 and the risk-associated H402 variants of CFH. The H402 risk variant binds less efficiently to certain extracellular moieties and oxidation epitopes.^25^^–^^29^ However, it remains unknown how or whether these interactions contribute to risk for AMD. We developed and characterized a mouse model of AMD that expresses the human H402 variant of CFH (*CFH-H/H*) to study the role of the H402 CFH risk variant in AMD pathobiology in vivo*.*^30^ This mouse develops an AMD-like phenotype when challenged with other known AMD risk factors, including advanced age and a high-fat, cholesterol-enriched (HFC) diet.^31^ Importantly, this AMD-like phenotype correlates with changes in lipoprotein levels in the RPE/BrM, and the AMD-like pathology in *CFH-H/H* mice is not observed in age-matched control mice expressing the normal human Y402 variant (*CFH-Y/0)* fed an HFC diet.^31^ This demonstrates a functional consequence of the Y402H polymorphism that promotes an AMD-like pathology in vivo in aged *CFH-H/H* mice fed an HFC diet.

Together, these findings implicate the APC in the development and progression of AMD. Furthermore, this evidence supports targeting CFH as therapy for AMD, especially through augmentation of the protective form of CFH.^32^ CFH is synthesized in both the RPE^32^ and liver, but the relative contribution from each of these tissues to ocular complement regulation is still not completely understood. The human *CFH* gene also encodes a splice variant, factor H-like 1 (FHL-1). FHL-1 consists of the N-terminal third of the protein and retains the domains necessary for complement regulation^33^^,^^34^ as well as the Y402H polymorphism in SCR 7^10^ (Fig. 1A). Due to its smaller size, the cDNA coding for FHL-1 readily fits into an adeno-associated virus (AAV) vector and was used for one of the truncated AAV vector constructs we generated (Fig. 1B). However, it lacks other domains in the C-terminus that help CFH bind extracellular surfaces and C3 (Fig. 1A).^35^^–^^38^ The absence of these domains has been implicated in disease risk in some individuals.^14^ CFH is a large molecule (1231 AA, 139 kDa) with a serum concentration of around 500 µg/mL^39^ that cannot passively diffuse across BrM, whereas the smaller FHL-1 (449 AA, 51 kDa) can.^40^ Previous studies have concluded that local ocular production of CFH, rather than systemic complement activity, underlies the most important contributions to AMD risk.^41^^,^^42^ However, these experiments did not reveal the contribution of FHL-1 relative to full-length CFH and did not use animal models. To answer this question, we examined the function of CFH and FHL-1 in the murine eye in the *Cfh* knock-out (*Cfh*^–/–^) mouse^21^^,^^43^ following either sustained local or systemic expression of AAV constructs carrying the cDNA for human *CFH* or *FHL-1*. A full-length CFH construct could not be expressed, likely due to AAV packaging limits.^44^ Accordingly, a truncated CFH (tCFH) was designed that eliminates two SCRs from the protein for a total of 18 SCRs while retaining the N- and C-terminals and their regulatory functions (Fig. 1A). Herein we show that tCFH regulated the APC in both the circulation and in the eye, but, surprisingly, FHL-1 only displayed complement regulatory function in the circulation. Both were also found in the posterior eye following expression from the liver. These findings will help refine and optimize the design of CFH-based complement therapies and support a role for the CFH C-terminal SCRs in ocular complement regulation.

## Methods

### Generation of Plasmids and AAV Constructs

The *tCFH* cDNA was designed to encode all 20 of the SCRs in full-length CFH except SCRs 16 and 17 (Fig. 1) and was sequence verified. Both *tCFH* and *FHL-1* cDNAs code for the normal Y402 AA tyrosine at the 402 position (Y402). The CBA–FHL-1 and the smallCBA (smCBA)–tCFH plasmids were generated and packaged into AAVs by Applied Genetic Technologies Corporation (Alachua, FL, USA). The thyroxine-binding globin (TBG)–FHL-1–woodchuck hepatitis virus post-transcriptional regulatory element (WPRE) and TBG–tCFH plasmids with flanking AAV inverted terminal repeat sequences were generated by VectorBuilder (Chicago, IL, USA) (Fig. 1B). The plasmids were packaged into AAV8 capsids in a lab at Duke University (author AA).

### In Vitro Tests

Plasmids coding for full-length CFH (flCFH), FHL-1, tCFH, or a green fluorescent protein (GFP) control were transfected into human embryonic kidney (HEK) 293 cells using Lipofectamine LTX Reagent in Gibco Opti-MEM medium containing no serum (Thermo Fisher Scientific, Waltham, MA, USA). Supernatants were collected 24 hours post-transfection, and the C3b cleavage activity of the different CFH variants was determined by incubating C3b, CFI, and the supernatant together for 1 hour at 37°C. Purified CFH was used as a positive control. All complement assay reagents were obtained from Complement Technology (Tyler, TX, USA). The resulting cleavage products were analyzed by Jess ProteinSimple western assays (Bio-Techne, Minneapolis, MN, USA).

For hemolysis assays, normal human serum (NHS) was diluted to 40%, then 50 µL of diluted NHS was incubated with 50 µL of testing solution in a 96-well plate. Supernatant of HEK293 cells transfected with and expressing flCFH, tCFH, or FHL-1 were used to test their ability to regulate the complement system. Sheep red blood cells (RBCs) were washed with PBS before being resuspended in gelatin veronal buffer with 0.15-mM Ca^2+^ and 0.5-mM Mg^2+^ at a concentration of 8.33E7 cells/mL. Next, 30 µL of RBC suspension was added to each well of the 96-well plate and incubated at 37°C for 30 minutes. Plates were then centrifuged, and 100 µL of supernatant was transferred to a new 96-well plate. Absorbance was measured between 410 and 570 nm, and the percent hemolysis was calculated. All reagents were obtained from Complement Technology.

### Animals and Viral Injections

*Cfh* ^–/–^ mice^21^^,^^43^ were housed and maintained in accordance with the Institutional Animal Care and Use Committee at Duke University under an approved protocol (#A075-21-03) in adherence with the ARVO Statement for the Use of Animals in Ophthalmic and Vision Research. For injections, *Cfh*^–/–^ mice (*n* = 6 to 9 per group) were anesthetized using an isoflurane/oxygen mixture (2%–3% in O_2_) in an induction chamber and maintained (1.5%–2.0% in O_2_) using a nose cone on a surgery stage. Each pupil was dilated using drops containing 1.25% phenylephrine and 0.5% tropicamide (1:1 mixture of 2.5% phenylephrine obtained from Akorn Pharmaceuticals, Lake Forest, IL, USA; and 1.0% tropicamide obtained from Bausch & Lomb, St. Louis, MO, USA). The cornea was anesthetized using drops of 0.5% proparacaine hydrochloride (Akorn). Using an ocular surgery microscope with foot pedal control of magnification and focus (OPMI S5; Zeiss Microscopy, Oberkochen, Germany), we used an injection procedure adapted from Park et al.^45^ Briefly, a sclerotomy hole was made in a temporal–inferior location just below the limbus using a 0.5-inch 30-gauge needle (305106; Becton, Dickinson, Franklin Lakes, NJ, USA). For subretinal injections, we used a 10-µL Hamilton syringe retrofitted with a spring-loaded lever-controlled plunger (7642-01; Hamilton Company, Reno, NV, USA) to allow for single-handed positioning and injection. One microliter of vector suspension [1 × 10^10^, 1 × 10^11^, or 1 × 10^12^ vector genomes [vg]/mL; 1E10–1E12) vg/mL) was delivered with the above syringe via a 0.375-inch 33-gauge blunt Hamilton needle (7803-05; Hamilton Company) extending across the vitreous behind the lens and penetrating through the retina in a nasal–superior location. Solution was delivered over 5 seconds, and the needle was held in place for 10 seconds after the injection was finished to allow for the solution to spread subretinally and thus pressure to decrease. This minimized reflux into the vitreous on needle withdrawal. Only a single (left) eye was injected in each mouse, leaving the contralateral eye as a control. For lateral tail vein injections, mice were restrained, and the veins on their tail were dilated with warm water. Then, 200 µL of solution containing 1E12 (FHL-1) or 4E12 (tCFH) vg in sterile saline were delivered via a 28-gauge insulin syringe (329424; Becton, Dickinson).

### Electroretinograms

Electroretinograms were recorded as previously described. Briefly, mice were dark adapted for 3 hours before being anesthetized with ketamine (100 mg/kg) and xylazine (10 mg/kg) and their eyes dilated with topical tropicamide (0.5%) and phenylephrine (1.25%). Measurements were made using and Espion E^2^ system (Diagnosys Systems, Orlando, FL, USA) with increasing flash intensities. B-wave amplitudes were measured from the preceding A-wave minimum to the B-wave peak and plotted versus flash intensity.

### Western Blots

Two months (subretinally injected animals) or 6 to 12 months (tail vein-injected animals) after injection, the *Cfh*^–/–^ mice were sacrificed and samples collected. Samples were prepared and Western blots run as we have previously described.^31^ Briefly, blood was collected via submandibular vein bleeds in tubes containing EDTA and spun to collect plasma. Mice were euthanized with CO_2_ and perfused with PBS before enucleation, the anterior segment and lens were removed and discarded, and the posterior segment was separated into retina and eyecup (RPE–choroid–sclera). Eyecup and retina lysates were made using RIPA buffer (89901; Thermo Fisher Scientific) containing cOmplete Mini EDTA-free protease inhibitor cocktail tablets (11836170001; Roche, Basel, Switzerland) and quantified using the Pierce BCA Protein Assay Kit (23227; Thermo Fisher Scientific). Plasma samples were diluted in a 10% 4× stock of XT Sample Buffer (1610791; Bio-Rad, Hercules, CA, USA). Western blots were run with non-reducing conditions using precast 10% Criterion XT Bis-Tris polyacrylamide gels (3450112; Bio-Rad) and MOPS buffer (1610788; Bio-Rad) with equal amounts of total protein of lysates loaded in each well as determined by the bicinchoninic acid (BCA) assay for eyecup and retina samples, and with an equal volume of diluted plasma for plasma samples. After transfer, the nitrocellulose membranes were blocked with 10% bovine serum albumin and incubated overnight with goat anti-CFH (A312; QuidelOrtho, San Diego, CA, USA) or goat anti-factor B (FB; Kent Laboratories, Bellingham, WA, USA) antibodies. Washes were done with Tris-buffered saline with 0.1% Tween 20 detergent (TBST). Peroxidase-conjugated bovine anti-goat secondaries (805-035-180; Jackson ImmunoResearch Labs, West Grove, PA, USA) and detected via chemiluminescence after the addition of Pierce ECL (32132; Thermo Fisher Scientific) with a ChemiDoc Imaging System (Bio-Rad). Densitometry was performed using ImageJ software (National Institutes of Health, Bethesda, MD, USA).

### Statistical Analysis

Data were plotted as individual points with error bars representing the standard deviation (SD). Unpaired *t*-tests were used for single comparisons and Tukey's range test was used for multiple comparisons. Mean value statistical significance was set at *P* < 0.05.

## Results

### CFH AAV Construct Design and Validation

**Figure 2.**
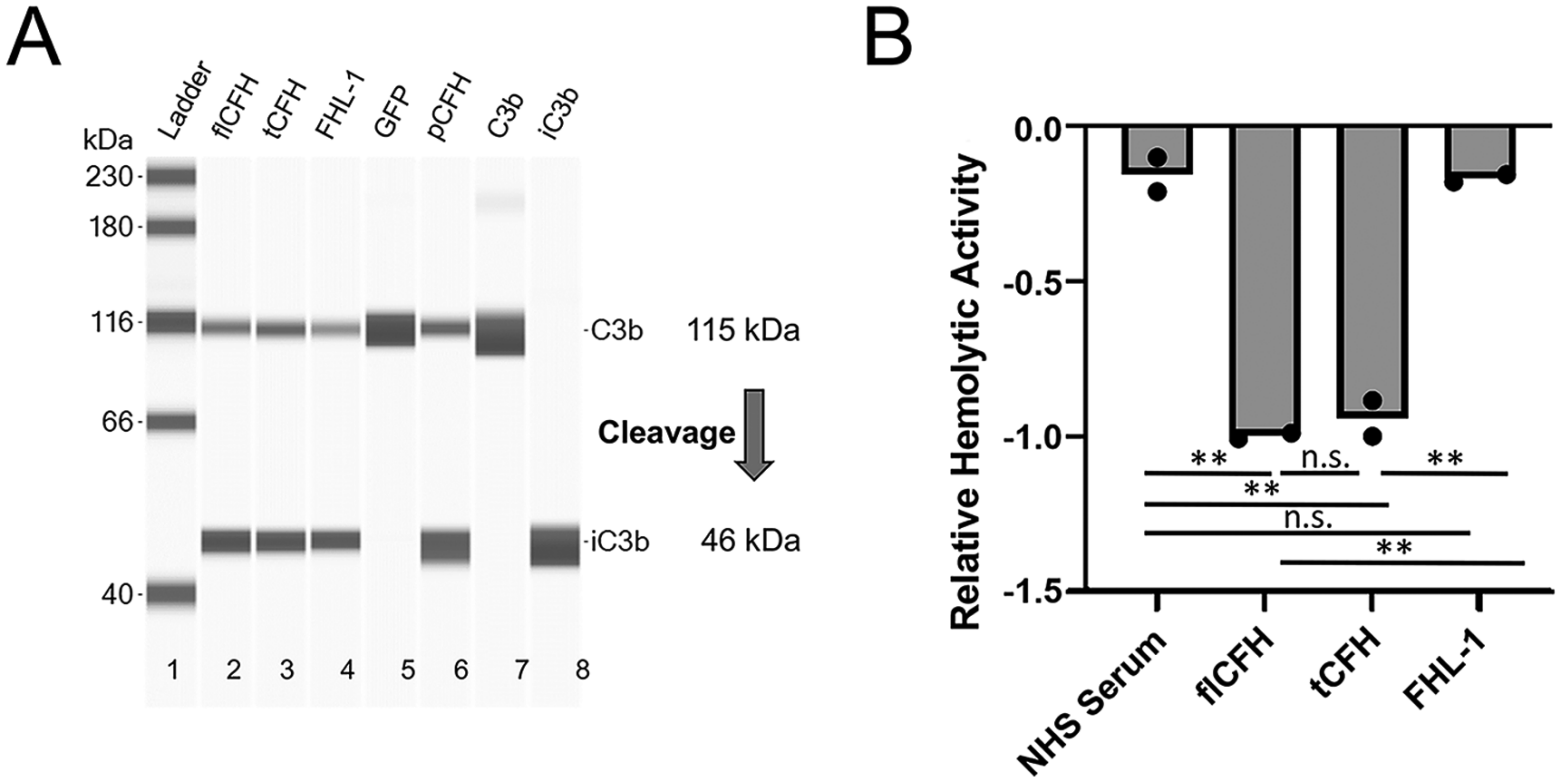
In vitro assay of activity of truncated CFH proteins. (**A**) The cofactor activity of FHL-1 and tCFH was tested using the supernatant from transfected HEK293 cells incubated with C3b and CFI. The reaction solution was then run on a capillary Western blot. tCFH and FHL-1 (lanes 3 and 4, respectively) showed similar amounts of C3 cleavage compared to supernatant from cells transfected with plasmids containing full-length CFH (flCFH, lane 2) or purified CFH (pCFH, (lane 6). No cleavage was observed in a GFP transfected control (lane 5). Control C3b and iC3b are shown in lanes 7 and 8, respectively. (**B**) Relative hemolytic activity on sheep red blood cells (RBCs) of the constructs compared to a normal human serum (NHS) control. Decreased hemolytic activity in the presence of the constructs was normalized to flCFH. tCFH showed activity nearly as robust as for flCFH. FHL-1 showed significantly lower activity similar to the NHS serum control.

Schematics of the SCRs in full-length CFH, FHL-1, and tCFH proteins used in this study are shown in Fig. 1A. FHL-1 is an alternative splice product of *CFH* in humans spanning the first seven SCRs. Due to inclusion of the first four N-terminal domains, FHL-1 is capable of binding fluid-phase C3b and regulating the alternative-pathway C3 convertase. At the C-terminus, FHL-1 is comprised of four unique amino acids.^33^^,^^36^ tCFH spans SCRs 1 to 15 and 18 to 20 and is missing two SCRs near the C-terminal end, 16 and 17. SCRs 16 and 17 demonstrated low-affinity dimerization (*K_D_* = 5 µm) when expressed as a fragment, which essentially disappeared in full-length CFH.^46^ These SCRs have no other published function in the regulation by CFH of the complement pathway or in its binding to cell surfaces.^46^ Previous experiments in our lab showed no expression using AAVs packaged with the full-length *CFH* genes, whereas the 10% shorter tCFH had readily detectable expression, as shown below. Regulatory function of the constructs was confirmed in vitro prior to in vivo tests (Fig. 2). Both FHL-1 and tCFH showed similar cofactor activity for CFI-mediated cleavage of C3b to iC3b compared to flCFH (Fig. 2A). FHL-1 showed hemolytic activity levels similar to those for NHS but with far less reduction in hemolytic activity compared to flCFH and tCFH (Fig. 2B). This finding suggests that FHL-1 does not regulate complement on the surfaces of the RBCs as efficiently as flCFH and tCFH. The hemolytic activity of tCFH was comparable to that of flCFH.

### tCFH Regulates FB Cleavage Following Ocular Expression But FHL-1 Does Not

The effect of local expression of exogenous CFH constructs on the APC in *Cfh*^–/–^ mouse eyes was examined 8 weeks after administration of 1 µL of an AAV construct suspension by subretinal injection in one eye, with the contralateral eye serving as a non-injected control. Specifically, viral doses of 1E7 to 1E9 vg of the FHL-1 AAV (AAV2tYF–CBA–FHL-1) or 1E9 vg of tCFH AAV (AAV2tYF–smCBA–tCFH) were delivered to the RPE by subretinal injection (see Fig. 1 for a schematic of the constructs). ERGs were recorded 8 weeks after the injection of the AAV CFH constructs, and the B-waves were plotted for each mouse at each viral titer (Supplementary Fig. S1). Visual function was only attenuated following injection of the viruses at the highest viral titers tested (1E9) resulting in lower B-wave amplitudes. In addition, this effect appeared to be independent of the construct being expressed, as AAVs expressing FHL-1 or tCFH had a similar decrease in B-wave amplitude as a 1E9-vg injection of control AAV expressing GFP (AAV2tYF–CBA–GFP) compared to paired non-injected contralateral eyes.

**Figure 3.**
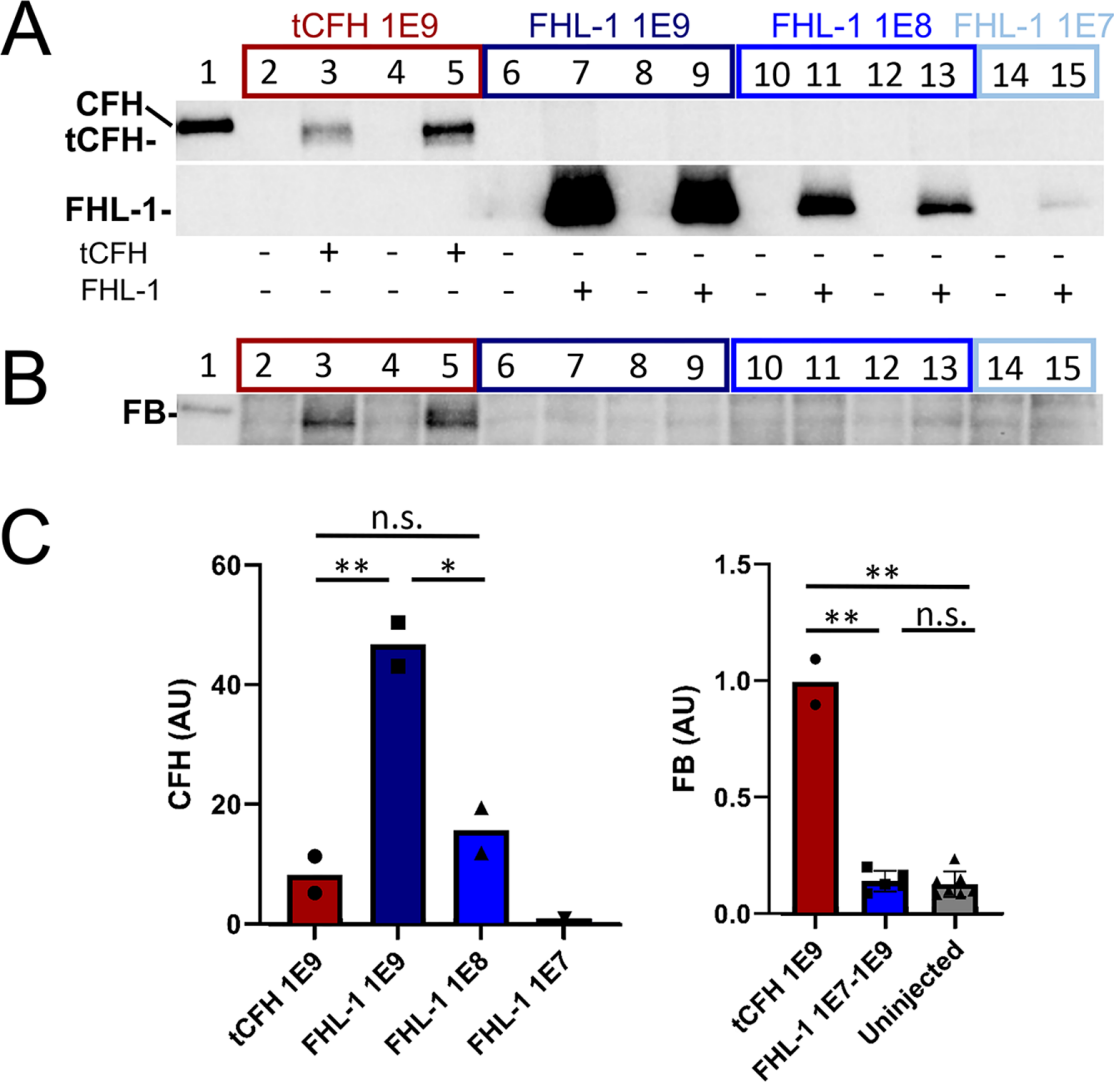
Posterior eyecup expression of endogenous FB in *Cfh*^–/–^ mice is CFH construct dependent. CFH (**A**) and FB (**B**) immunoblots of eyecup (RPE/BrM/choroid/sclera) lysates isolated from *Cfh*^–/–^ mice following subretinal injections with AAVs expressing tCFH (lanes 3 and 5, *red box*) or FHL-1 (lanes 7, 9, 11, 13, and 15; *dark blue*, *blue*, and *light blue boxes*). Corresponding dose vector genomes (vg) of each subretinal injection are shown on the *x*-axis of the graphs in **C**. The non-injected control contralateral eyecup lysates (–) were loaded in the even-numbered lane to the left of each injected eyecup lysate (+). Lane 1 is a positive control for full-length CFH (*CFH H/H* mouse plasma). (**C**) Densitometric analysis of immunoblots in **A** and **B**. The relative protein levels measured by densitometry are depicted in the bar graphs (CFH, *left panel*; FB, *right panel*). The CFH values are normalized to the single eye with FHL-1 from the lowest dose group (1E7 vg, lane 15, *light blue box*). Even though the levels of FHL-1 are about sixfold higher than tCFH at an equivalent viral dose, only the two eyecup lysates from eyes expressing the tCFH have intact FB. *P < 0.05; **P < 0.01.

The amount of the FHL-1 and tCFH in lysates of retina and eyecups was measured by Western blot analysis. Injected eyes with detectable tCFH or FHL-1 are shown in Fig. 3A. Two out of three AAV2tYF–smCBA–tCFH-injected eyes showed expression for the tCFH-injected animals. Two out of three AAV2tYF–CBA–FHL-1–injected eyes showed expression of FHL-1 in the two higher titer groups of animals, and only one out of three showed expression of the lowest dose. Densitometric quantification showed a correlation of expression in the *Cfh*^–/–^ eyecup with viral dose of the FHL-1 AAV, with the average expression of the highest dose (1E9 vg) statistically higher than all other groups (Fig. 3C, left panel). tCFH expression was nearly six times lower than that of FHL-1 at the same viral titer (1E9 vg). This is likely due to the larger size of the *tCFH* cDNA, which requires use of a smCBA promoter to fit within the AAV, instead of the stronger full-length CBA promoter used with our FHL-1 construct.

FB was used as a readout for the ability of each construct to regulate the APC. As part of the activation of the alternative pathway, FB binds C3b and is cleaved into Bb and Ba. The Bb remains bound to C3b to form the C3 and C5 convertases.^22^ In *Cfh*^–/–^ mice, the APC is constantly activated, resulting in complete consumption of cleavable complement proteins, including FB. This effectively results in functional depletion of C3 and FB.^30^^,^^47^ We have previously shown a direct correlation between amounts of full-length endogenous CFH expression and the amount of uncleaved FB.^30^^,^^31^ Eyecup lysates prepared from FHL-1–injected eyes showed no intact FB, even in lysates from eyes with the highest FHL-1 concentration (Fig. 3B). In contrast, FB was detectable in *Cfh*^–/–^ eyecup lysates with tCFH expression, despite the significantly lower concentrations (∼sixfold lower) relative to FHL-1 levels.

### Lack of Correlation Between tCFH Expression and FB Regulation

**Figure 4.**
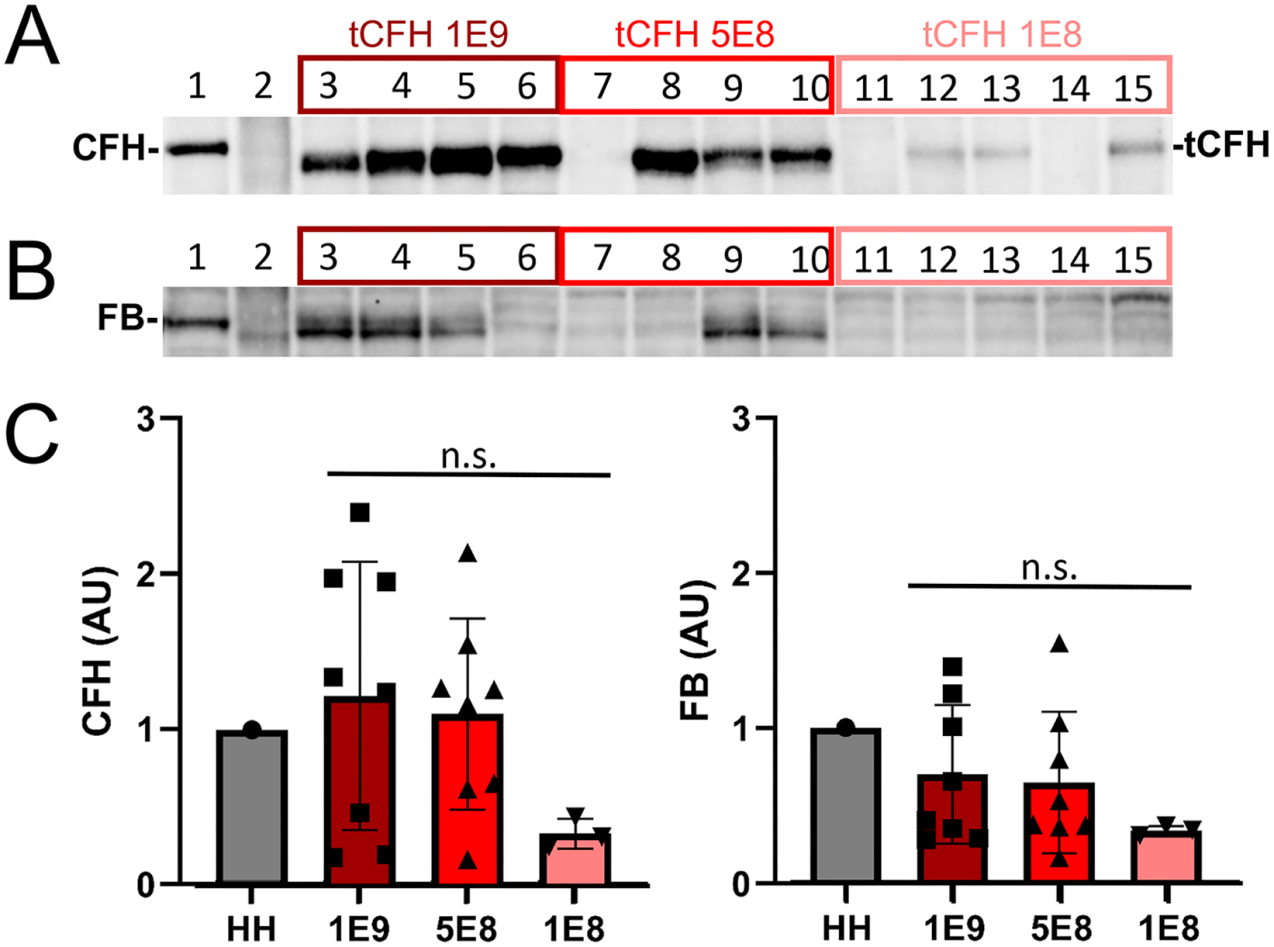
Dose-dependent regulatory function of tCFH. CFH (**A**) and FB (**B**) immunoblots of eyecup lysates isolated from *Cfh*^–/–^ mice following subretinal injections at three different doses with AAVs expressing tCFH: 1E9 vg (*dark red box*), 5E8 vg (*red box*), and 1E8 vg (*pink box*). Lane 1 is a positive control for full-length CFH, *CFH H/H* (HH) mouse eyecup lysate. Lane 2 was loaded with the molecular weight marker. (**C**) Densitometric analysis of immunoblots in **A** and **B** using only mice that showed expression of their construct. Eight out of nine mice showed expression for the two higher viral titers, whereas only three out of nine showed expression for the lower titer. The relative amount of expression measured by densitometry is depicted in the bar graph. The values are normalized to the control lysate in lane 1. The two highest dose groups show similar expression of CFH. Although many of the high-expression eyes showed an intact FB band, there is not a direct correlation between expression of the construct and its regulatory activity.

In a separate experiment, 1E8, 5E8, or 1E9 vg of the tCFH expressing AAV (AAV2tYF–smCBA–tCFH; no SCR16 or 17) was subretinally injected into *Cfh*^–/–^ mice (*n* = 9). Eight weeks later the amounts of tCFH in eyecup lysates were measured on Western blots (Fig. 4A). Although there was a twofold difference between the two highest doses injected (1E9 and 5E8 vg), tCFH levels detected in the eyecup lysates were not statistically different. Only mice that showed expression were included in the densitometric analysis. Eight out of nine mice showed expression and had received one of the higher two doses. In contrast, tCFH was detectable in the eyecup lysates of only three out of nine mice injected with the lowest titer (1E8 vg) (Fig. 4C). Comparing AAV-injected *Cfh*^–/–^ eyecup lysates with detectable tCFH revealed no direct correlation between the quantities of tCFH and FB detected (Fig. 4B). Some eyes with high expression of tCFH showed little or no intact FB. There was a similar pattern of tCFH levels detected on Western blots of retina lysates (Supplementary Fig. S2A), but no FB was detected in any retina sample (Supplementary Fig. S2B). This is to be expected, as there is no CFH or FB in the neural retina of control *CFH H/H* mouse (Supplementary Fig. S2).

### Systemic FHL-1 and tCFH Expressed in Liver Differentially Regulates Complement in Plasma

**Figure 5.**
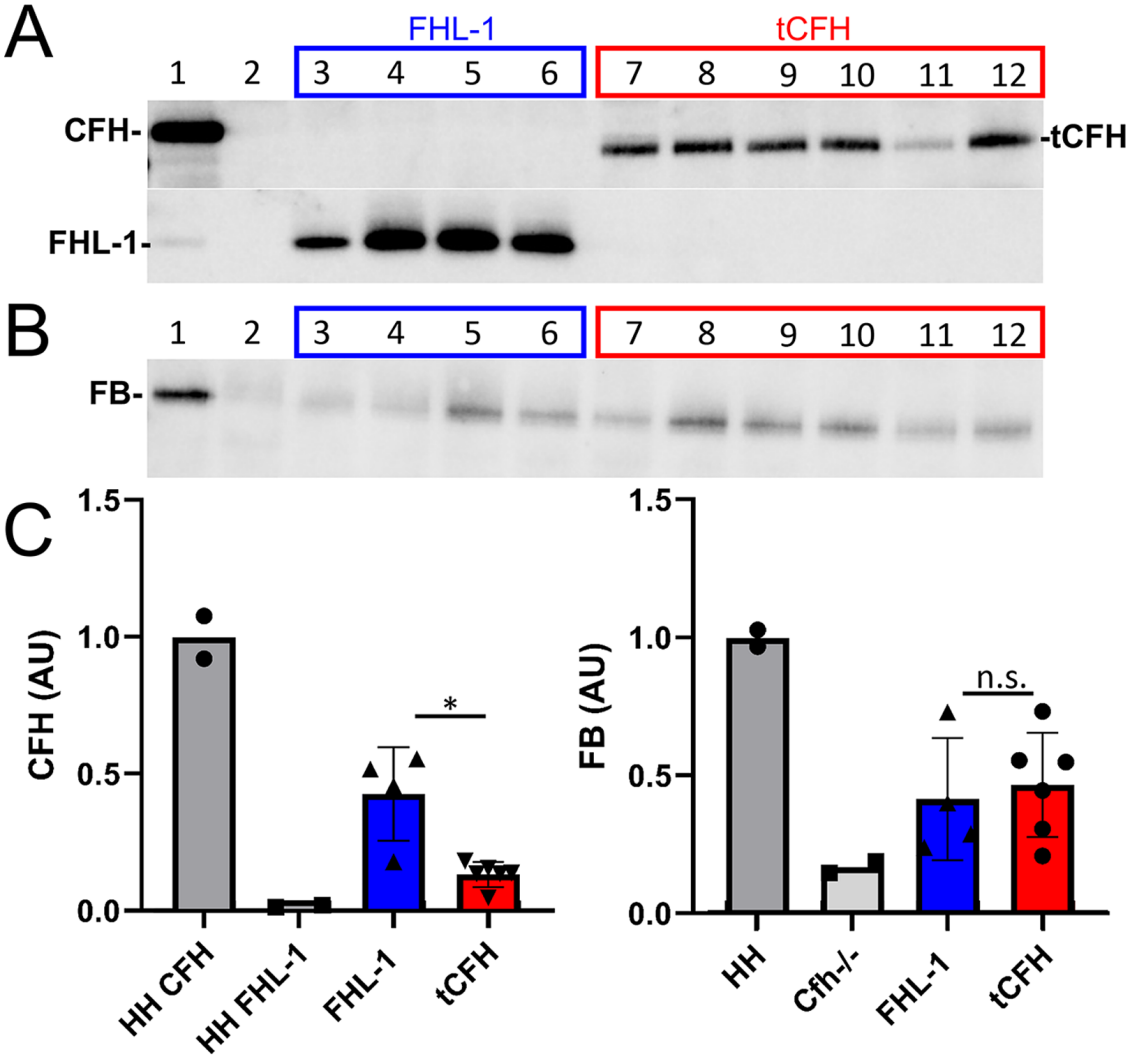
Both FHL-1 and tCFH regulate complement activation in plasma. CFH (**A**) and FB (**B**) immunoblots of equal volumes of plasma collected from mice following tail vein injection of AAVs containing FHL-1 (*blue*) or tCFH (*red*), with expression driven by a liver-specific promoter. Lane 1 is a positive control for full-length CFH (*CFH H/H* plasma). Lane 2 was loaded with plasma from a non-injected *Cfh*^–/–^ animal. (**C**) Densitometric analysis of immunoblots in **A** and **B**. The relative amount of expression measured by densitometry is depicted in the bar graph. The values are normalized to the endogenous CFH (left graph) or FB (right graph) in control plasma in lane 1 (HH). FHL-1 shows higher expression than tCFH, but there is no statistical difference in the amount of FB between the two AAV-injected groups. * *P* < 0.05.

The main source of circulating endogenous CFH found in the bloodstream comes from the liver.^41^^,^^48^ Therefore, AAV constructs were designed to express FHL-1 and tCFH in the liver to achieve systemic protein circulation (see Fig. 1 for a schematic of these constructs). Specifically, *Cfh*^–/–^ mice were injected via the tail vein with AAV8–CFH constructs driven by the liver-specific thyroxine binding globulin (TBG, also known as SERPINA7) promoter. The FHL-1 expressing AAV (AAV8–TBG–FHL-1) was delivered at a dose of 1E12 vg in 200 µL, whereas tCFH–AAV (AAV8–TBG–tCFH) was delivered at 4E12 vg in 200 µL to account for lower expression of tCFH constructs detected in the subretinally injected samples. Equal volumes of plasma were run from each mouse on a Western blot. Plasma from *CFH H/H* mice, which express both flCFH and FHL-1, was used as a control.^30^^,^^31^ Endogenous plasma FHL-1 levels were much lower (approximately 60-fold lower) than CFH levels in *CFH H/H* mice (Figs. 5A, 5C). This is consistent with reported human plasma levels of FHL-1 where the molar ratio of CFH to FHL-1 is approximately 40:1.^49^ In the virally transduced *Cfh*^–/–^ mice, the protein levels detected for the two constructs was statistically different (*P* = 0.04). Circulating levels of FHL-1 were statistically higher than the levels of tCFH and approached about half the levels of full-length CFH in *CFH H/H* mice (Figs. 5A, 5C, left panel).

The function of the expressed truncated CFH proteins was again evaluated by measuring levels of intact FB on Western blots of the plasma (Fig. 5B). Mice expressing tCFH and FHL-1 showed some rescue of plasma FB (Fig. 5B) compared to non-injected *Cfh*^–/–^ animals, in contrast to the lack of FB in FHL-1–expressing eyecups following subretinal injection described in the previous results section. In addition, there was no statistical difference in the amount of intact FB in the plasma of tCFH and FHL-1 expressing mice (*P* = 0.71) despite the higher levels of FHL-1 compared to tCFH, suggesting the tCFH may be working more efficiently than FHL-1 in plasma (Fig. 5B).

### Liver-Expressed Truncated CFHs Accumulate in the Eye

**Figure 6.**
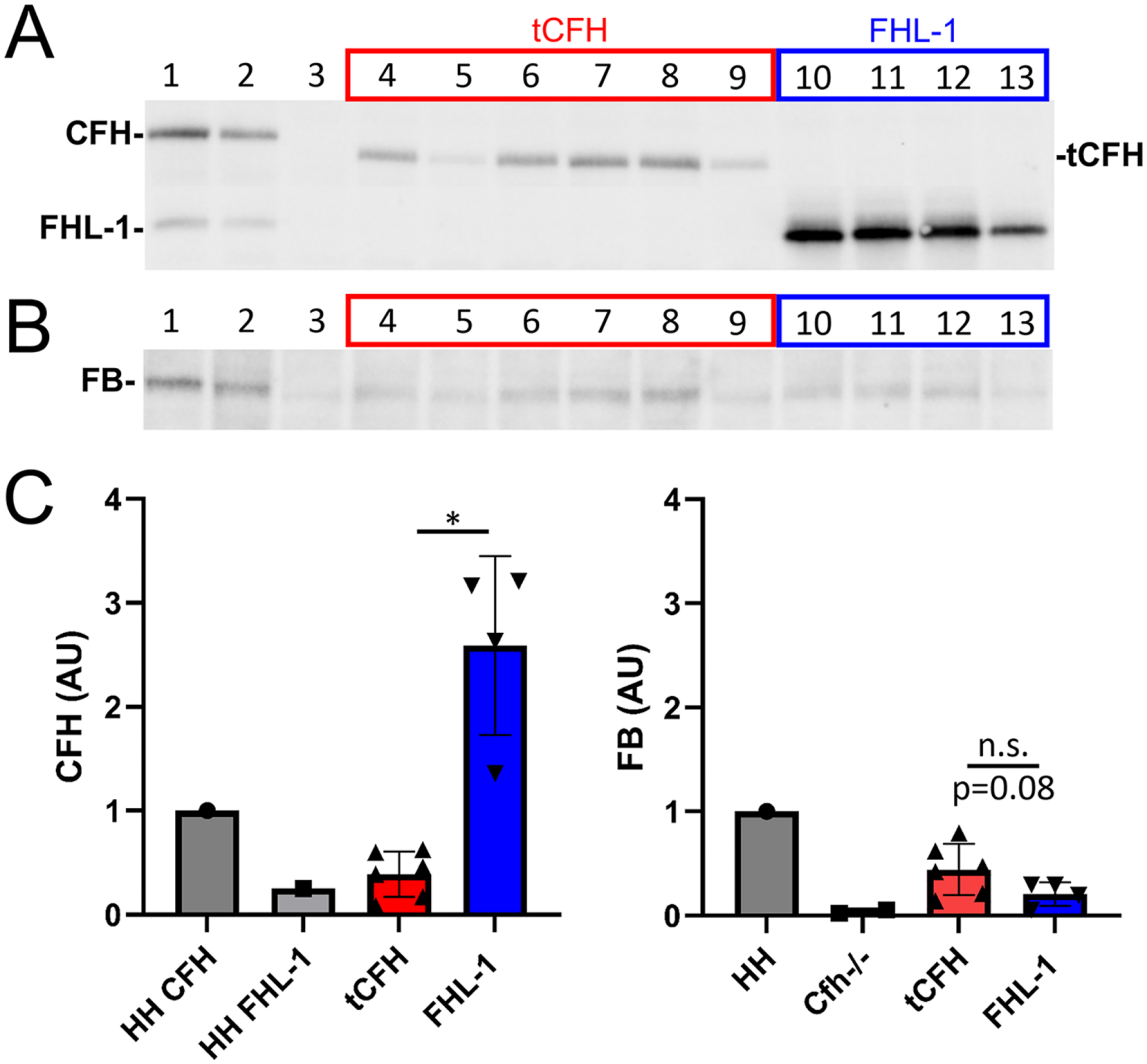
Localization and function of liver-expressed constructs in the eye. CFH (**A**) and FB (**B**) immunoblots of eyecup lysates isolated from *Cfh*^–/–^ mice following tail vein injection of AAVs containing FHL-1 (*blue*) or tCFH (*red*). Lane 1 is a positive control for full-length CFH (*CFH H/H* lysate), and lane 2 is a hemizygous CFH mouse (*CFH H/0*), which expresses half as much CFH as a *CFH H/H* mouse.^31^ Lane 3 was loaded with lysate from a non-injected *Cfh*^–/–^ mouse. (**C**) Densitometric analysis of immunoblots in **A** and **B**. The relative amount of expression measured by densitometry is depicted in the bar graph. CFH, *left panel*; FB, *right panel*. The values are normalized to the control *CFH H/H* eyecup lysate in lane 1. FHL-1 accumulated in the eyecups of liver-expressing mice but showed minimal FB. *P < 0.05.

FHL-1 and tCFH were readily detectable in the eyes of tail vein-injected mice by Western blot (Figs. 6A, 6C, left panel). Densitometry of the CFH Western blots showed that FHL-1 levels were higher in the eyecups relative to CFH in control eyecups (Fig. 6C, left panel) than in plasma relative to CFH in control plasma. The amount of endogenous full-length CFH in *CFH H/H* plasma was higher compared to exogenous FHL-1 expression in *Cfh*^–/–^ plasma, but this was reversed in the eyecups, demonstrating increased accumulation of FHL-1.

### Differences in Ocular Complement Regulation Between Liver-Expressed Constructs

Comparison of intact FB in the eyecups of *Cfh*^–/–^ animals expressing truncated CFH from their livers showed detectable amounts above background in both the FHL-1–injected and tCFH-injected mice (Figs. 6B, 6C, right panel). Levels of FB in the eyecups of tCFH tail vein-injected animals were not statistically different compared to FB levels in eyecups of FHL-1 tail vein-injected animal, even though the tCFH FB was trending approximately twofold higher (*P* = 0.08) (Fig. 6C, right panel). This reflects the finding in the plasma of these animals where, despite higher FHL-1 levels relative to tCFH, the restoration of FB levels is similar.

## Discussion

AMD risk is associated with increased complement activation^9^ and polymorphisms in CFH.^10^^–^^14^^,^^19^ We set out to test the therapeutic potential of truncated *CFH* constructs to restore complement regulation in the eyes of *Cfh*^–/–^ mice using AAVs to express the cDNAs locally following subretinal injection, and systemically following tail vein injection with a liver-specific promoter. The two CFH transgenes encoded a truncated CFH (tCFH), which retained both the N- and C- termini of full-length CFH, or the human splice variant FHL-1, which consists of the N-terminal third of the protein. Although both FHL-1 and tCFH showed similar cofactor activity for in vitro cleavage of C3b to iC3b, FHL-1 showed less regulation of hemolytic activity for RBCs compared to both tCFH and full-length CFH. Similarly, we found that high levels of ocular FHL-1 expression were unable to prevent unregulated complement activation as measured by cleavage of FB, whereas ocular tCFH appeared to regulate complement despite lower levels of expression. In contrast to the lack of FB in *Cfh*^–/–^ eyecups expressing FHL-1 following subretinal injection, FB was detected in the plasma of *Cfh*^–/–^ mice following tail vein injection and liver expression of FHL-1. The amounts of both FHL-1 and tCFH were higher in the eyecup than in the circulation following liver expression compared to endogenous levels of CFH in the *CFH H/H* mouse, but there was less FB in their eyecups compared to the plasma from the same animals.

The majority of the complement proteins found in the circulation are synthesized by the liver,^48^ but other cell types and tissues in the eye also express complement proteins, including the RPE^10^ and choroid.^42^ Our results are consistent with local production of CFH (but not FHL-1) being more important to complement regulation in the eye than circulating CFH derived primarily from the liver. Previous work has also largely concluded this without addressing the relative functions of FHL-1 and full-length CFH.^41^^,^^42^ For example, in a study of human liver transplant patients and their subsequent AMD risk, host production of local, ocular CFH was more important.^41^ Following an assessment of genotypes among patients and their donor liver tissue, the authors showed that circulating Y402 or H402 CFH protein was derived from the donor liver tissue, but the patient's AMD risk was only associated with the patient's own *CFH* genotype and their local, ocular CFH protein expression.^41^ It has been shown that the terminal product of complement activation, the membrane attack complex, accumulates in human eyes with age and was associated with the risk-associated Y402H CFH polymorphism.^10^^,^^50^^–^^53^ The back of the eye has a rich blood supply from the choroid and choriocapillaris, but, whereas the choriocapillaris is highly fenestrated on the RPE-facing side, the blood does not come into direct contact with the RPE cells themselves due to the presence of BrM. Clark et al.^40^ compared CFH and FHL-1 diffusion through BrM using explants and an Ussing chamber. They showed that FHL-1 could diffuse into BrM but CFH could not, leading to their hypothesis that FHL-1 was the more relevant splice variant for complement regulation in the posterior eye. Previous studies have established a potential link between FHL-1 and early-onset macular drusen (EOMD), a form of macular degeneration with similar presentation to AMD but appearing at a younger age.^54^^–^^56^ A review of published cases reports supports that approximately 80% of reported EOMD-related CFH variants occur within the first seven SCRs of CFH, which would therefore affect FHL-1 as well as full-length CFH.^56^ It has previously been observed that C-terminal mutations in CFH are more associated with development of atypical hemolytic–uremic syndrome, and N-terminal mutations are more associated with AMD and C3 glomerulopathy.^57^ However, there are also some mutations that occur C-terminal to the first seven SCRs that are associated with EOMD and AMD risk, such as the R1210C mutation in SCR 20.^14^^,^^56^

In the context of these studies, the inability of FHL-1 to regulate complement in the eye following subretinal injection of an AAV for FHL-1 comes as a surprise. FHL-1 has recently been reported to have activity similar to that of full-length CFH,^34^^,^^49^ although at least one previous publication has reported greatly diminished regulation by FHL-1 compared to CFH on the surface of RBCs.^33^ Our data support less efficient regulation by FHL-1 both in vitro*,* based on hemolytic activity on sheep RBCs, and in vivo, where there was no statistical difference in the amount of intact circulating FB in plasma following tail vein injections of liver-expressing AAVs for FHL-1 and tCFH, despite three times higher levels of FHL-1 compared to tCFH in the circulation. One reason for the differences in reported activity of FHL-1 in previous studies may be the measurement in solution versus on surfaces and the different surfaces that have been used.^33^^,^^34^^,^^49^ Mice lack endogenous FHL-1, but our results clearly show it is still functional when interacting with the rest of the mouse complement proteins in circulation. CFH has previously been shown to be more effective at binding self-cell surfaces and regulating complement compared to FHL-1, but this was only reported as less than a twofold difference.^49^ As there is far more than a twofold excess of FHL-1 compared to tCFH in the subretinally injected *Cfh*^–/–^ eyes with the highest viral dose, this is not consistent with our results. Whether this is a species difference (mouse vs. human) or a unique aspect of the posterior eye warrants further investigation.

The amount of tCFH relative to full-length CFH in *CFH H/H* controls in liver-expressing *Cfh*^–/–^ mice increased approximately threefold from the circulation to the eye. However, the ratio of intact FB in the eyecups of animals expressing systemic tCFH compared to eyecups of *CFH H/H* controls did not change compared to the plasma. This result was surprising and suggests that the systemic tCFH is not functioning optimally in the eyecup, which may be because it is too large to cross into BrM, as shown for full-length CFH by Clark et al.^40^ A recent paper using cultured choroidal endothelial cells showed that production of CFH by these cells was needed to protect the cultured cells from complement attack rather than extracellular sources.^42^ The decreased ability of systemic, circulating tCFH to regulate complement in the eye is consistent with the in vitro choroidal endothelial cell study and suggests that systemic tCFH may be unable to localize or accumulate in the appropriate spaces in the back of the eye. In contrast to circulating tCFH, the exogenous, locally expressed tCFH in subretinally injected *Cfh*^–/–^ eyes restored complement regulation in the eyecups. Drusen, which form within BrM in the inner collagenous layer, have been shown to contain complement proteins,^10^ despite an inability of most complement proteins to diffuse through BrM.^58^ This lends support to the idea that the differences in complement regulation by subretinally injected tCFH versus systemically supplied tCFH may be caused by local regulation of complement within BrM. The ocular *tCFH* cDNA expression was driven by a ubiquitous promoter, and the AAV2tYF capsid has good tropism for both RPE and photoreceptors.^59^ tCFH measured on Western blots of eyecup lysates from these animals may include tCFH secreted from photoreceptors and accumulating on the apical side of the RPE. However, there is no detectable FB in the *CFH H/H* retina, which may explain the absence of a direct correlation between the levels of tCFH and FB in the eyecup.

The current study does not investigate the potential non-canonical roles of CFH or FHL-1 outside their complement regulatory function. While it has been observed that FHL-1 is the predominant CFH isoform present in BrM,^40^ it has also been shown that other complement proteins are unable to diffuse into BrM, including C3.^58^ Therefore, it is possible that FHL-1 may have some other role in BrM. We have previously shown that CFH competes with lipoproteins for binding to the extracellular matrix,^43^^,^^60^ and mice expressing human CFH show differences in lipoproteins depending on the Y402H polymorphism.^31^ Other potential roles for CFH in AMD include clearance of immune cells following inflammation.^61^ Although it is clear from the data presented here that FHL-1 does not regulate complement in the mouse eye, these other potential roles of CFH and FHL-1 warrant further investigation.

One limitation of our study is the use of transretinal subretinal injections. A previous study reported reduced visual function following subretinal AAV injections, but this could be ameliorated by restricting AAV expression to photoreceptors using photoreceptor-specific promoters.^62^ Future studies will be required to determine if RPE-specific expression of these complement regulators is optimal. Comparing intravitreal to subretinal delivery of an AAV complement inhibitor construct revealed that complement activation in the eyecup was only achieved in eyes following subretinal injection.^63^ This is consistent with our observation of regulation following subretinal injection. Our findings suggest that systemic delivery was not as effective as subretinal injection for achieving ocular complement regulation, and the systemic strategy required far higher doses of virus. High doses of AAV delivered intravitreally have been shown to cause ocular inflammation,^64^^,^^65^ but these side effects are absent following subretinal delivery. Although subretinal delivery has certain disadvantages in the aging and diseased retina, this may still be the optimal delivery method for any human viral therapies in the treatment of AMD. Ultimately, AAV gene augmentation represents an important opportunity to harness the therapeutic potential of complement regulators, such as tCFH, in the treatment of AMD and complement dysregulation.

## Supplementary Material

Supplement 1
