# Supplementary material for: AAV Gene Augmentation of Truncated Complement Factor H Differentially Rescues Ocular Complement Dysregulation in a Mouse Model
Source: Invest Ophthalmol Vis Sci. 2023 Jul 20;64(10):25. doi: 10.1167/iovs.64.10.25 (PMC10365136; doi:10.1167/iovs.64.10.25)
Supplement: Supplement 1 [file iovs-64-10-25_s001.pdf]

**AAV Gene Augmentation of Truncated Complement Factor H Differentially Rescues Complement Dysregulation in a Mouse Model.**

Daniel Grigsby[1], Mikael Klingeborn[1,2], Una Kelly[1], Lindsey A. Chew[1,7], Aravind Asokan[3], Garth Devlin[3], Sharon Smith[4], Lisa Keyes[5], Adrian Timmers[6], Abraham Scaria,[4] and Catherine Bowes Rickman[1,7,*]

## Supplementary Figures

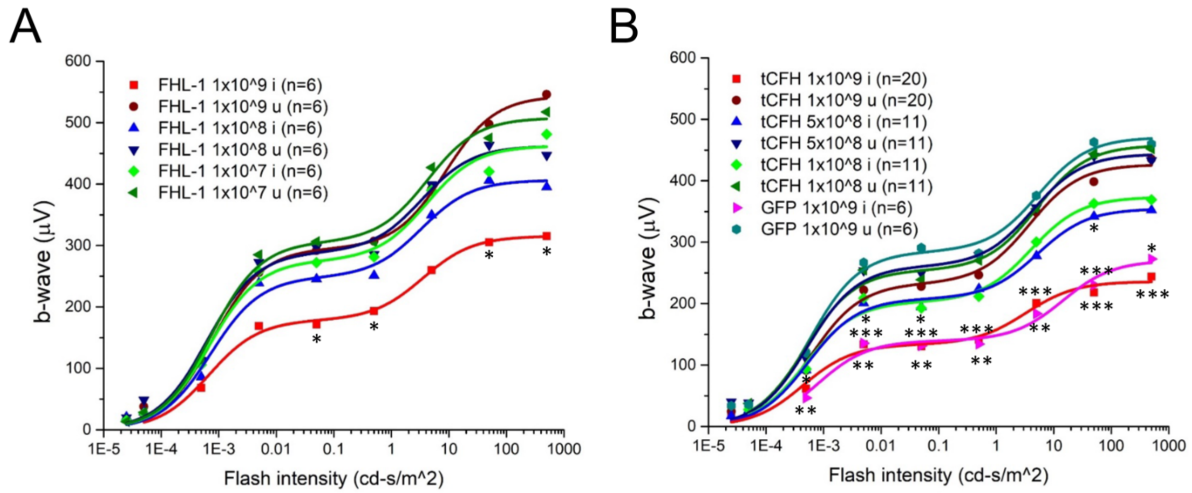


**Supplementary Figure 1. Visual function following subretinal injection of AAV viruses in *Cfh-/-* mice.** Scotopic ERG flash responses in *Cfh-/-* mice following subretinal injections of AAVs expressing FHL-1 (AAV2tYF-CBA-FHL1) (**A**) or tCFH (AAV2tYF-smCBA-tCFH) (**B**). Visual function was assessed by scotopic ERG and presented as fitted lines of the B-wave amplitude averages using the equation R = (Bmax1 × I/I + I1)/(Bmax2 × I/I + I2). (**A**) No differences in the scotopic ERG B-wave responses were observed in any of the uninjected contralateral eyes, *u*, or in the eyes of mice injected with the lowest FHL-1 titers (*FHL-1 1x10^7*, green trace or *FHL-1 1x10^8 i* blue trace). However, *Cfh-/-* mice injected with 10E9 FHL-1 (*FHL-1 1x10^9 i* red trace) develop significant attenuated ERG B-wave responses compared to uninjected. (**B**) No differences in the scotopic ERG B-wave responses were observed in any of the uninjected contralateral eyes, *u*. No differences were detected between uninjected mice and those in the lowest titer group (*tCFH 1x10^8 i,* green trace). Only three points were significantly different at the medium titer (*tCFH 5x10^8 i* blue trace). However, *Cfh-/-* mice injected with the highest tCFH titer (*tCFH 1x10^9 i* red trace) develop significant attenuated ERG B-wave responses compared to uninjected. Injections with a control GFP AAV, AAV2tYF-CBA-GFP at 10E9 (*GFP 1x10^9 i*, pink trace) also resulted in attenuated ERG B-wave responses, suggesting that this visual function loss is more likely due to high viral titer versus expressed protein. *, $P<0.05$; **, $P<0.01$; ***, $P<0.001$.

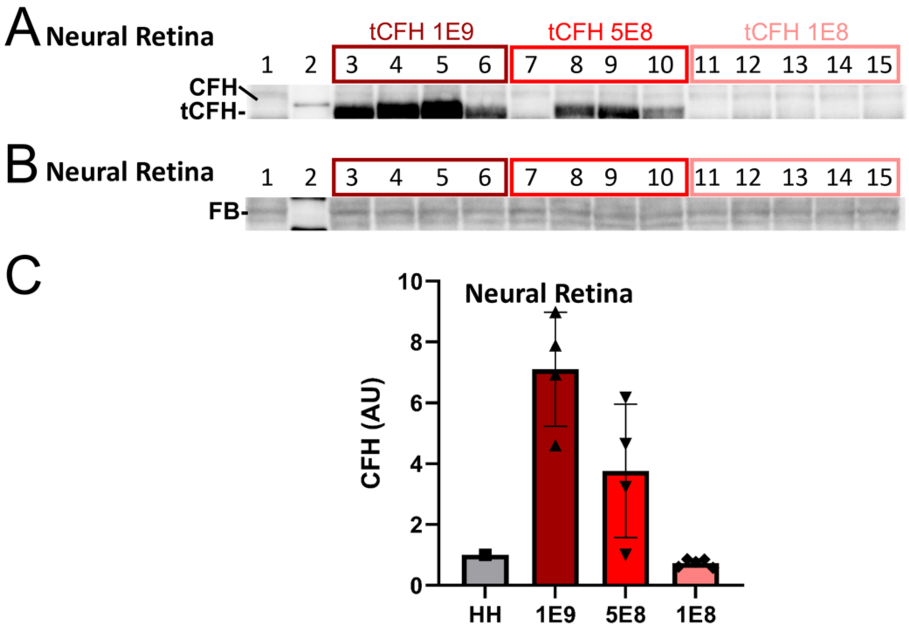


**Supplementary Figure 2. Expression and function of tCFH in neural retina after subretinal dosing.** CFH (**A**) and FB (**B**) immunoblots of neural retina lysates isolated from *Cfh-/-* mice following subretinal injections at three different doses with AAVs expressing tCFH: 1E9 vg (dark red, lanes 3-6), 5E8 vg (red, lanes 7-10), 1E8 vg (pink, lanes 11-15). Lane 1 is a positive control for full-length CFH [*CFH H/H* (HH) eyecup lysate]. Lane 2 was loaded with the molecular weight marker. There is a strong correlation between expression in the eyecup in **Fig. 3** and in the neural retina for tCFH in (**A**). In contrast, there is no CFH detectable in the neural retina of *CFH H/H* mice (Lane 1). No FB is seen in the neural retina in (**B**) in either control or injected eyes. (**C**) Densitometric analysis of immunoblots in (**A**). The relative amount of expression measured by densitometry is depicted in the bar graph. The values are normalized to the control *CFH H/H* lysate in Lane 1.
